# International survey among hepatologists and pulmonologists on the hepatic hydrothorax: plea for recommendations

**DOI:** 10.1186/s12876-023-02931-z

**Published:** 2023-09-12

**Authors:** Jean-François David Cadranel, Isabelle Ollivier-Hourmand, Jacques Cadranel, Thierry Thevenot, Honoré Zougmore, Eric Nguyen-Khac, Christophe Bureau, Manon Allaire, Jean-Baptiste Nousbaum, Véronique Loustaud-Ratti, Xavier Causse, Philippe Sogni, Bertrand Hanslik, Marc Bourliere, Jean-Marie Peron, Nathalie Ganne-Carrie, Thong Dao, Dominique Thabut, Bernard. Maitre, Nabil Debzi, Ryad Smadhi, Roger Sombie, Raimi Kpossou, Olivier Nouel, Julien Bissonnette, Isaac Ruiz, Mourad Medmoun, Sergio Negrin Dastis, Pierre Deltenre, Florent Artru, Chantal Raherison, Laure Elkrief, Tristan Lemagoarou

**Affiliations:** 1 Hepatogastroenterology and Nutrition Department GHPSO Boulevard Laennec, 60100 Creil, France; 2 Hepatogastroenterology department, CHU Caen, Caen, France; 3 Pneumology Department, Tenon, Paris, France; 4 Hepatology and Intensive Care Unit, Besançon, France; 5 Hepatogastroenterology department, Amiens, France; 6 Hepatogastroenterology department, Toulouse, France; 7 Hepatogastroenterology Department, La Pitié Salpétrière, Paris, France; 8 Hepatogastroenterology department, Brest, France; 9 Hepatogastroenterology department, Limoges, France; 10 Hepatogastroenterology department, Orléans, France; 11 Hepatology Department, Cochin, Paris, France; 12 Gastroenterology Practice, Montpellier, France; 13 Hepatogastroenterology department, Saint-Joseph, Marseille, France; 14 Hepatogastroenterology department, Bobigny, France; 15 Pneumology department, CHIC Créteil, Créteil, France; 16 Hepatology Department CHU Mustapha, Alger, Algérie Algeria; 17 Gastroenterology Department, CHU Yalgado Ouedraogo Ouagadougou, Ouagadougou, Burkina Faso; 18 Hepatogastroenterology Deparment, National Hospital and University Center Hubert Koutoukou Maga, Cotonou, Benin; 19 Hepatogastroenterology Department, St Brieuc, France; 20 Department of Hepatology and Liver Transplantation, University of Montreal Hospital, Montreal, Canada; 21 Hepatogastroenterology Department, Brussels, Belgium; 22 Hepatogastroenterology Department, Lausanne, Suisse Switzerland; 23 Pneumology department, Bordeaux, France; 24 Hepatogastroenterology department, Tours, France; 25 DIM, GHPSO, Creil, France

**Keywords:** Hepatic hydrothorax, Therapeutic pleural puncture, Albumin infusion, Spontaneous bacterial empyema, Talcage pleurodesis, Indwelling pleural catheter, TIPS, Liver transplantation, Portal hypertension, Cirrhosis

## Abstract

**Background:**

The Hepatic hydrothorax is a pleural effusion related to portal hypertension; its diagnosis and therapeutic management may be difficult. The aims of this article are which follows: To gather the practices of hepatogastroenterologists or pulmonologists practitioners regarding the diagnosis and management of the hepatic hydrothorax.

**Methods:**

Practitioners from 13 French- speaking countries were invited to answer an online questionnaire on the hepatic hydrothorax diagnosis and its management.

**Results:**

Five hundred twenty-eight practitioners (80% from France) responded to this survey. 75% were hepatogastroenterologists, 20% pulmonologists and the remaining 5% belonged to other specialities. The Hepatic hydrothorax can be located on the left lung for 64% of the responders (66% hepatogastroenterologists vs 57% pulmonologists; *p* = 0.25); The Hepatic hydrothorax can exist in the absence of clinical ascites for 91% of the responders (93% hepatogastroenterologists vs 88% pulmonologists; *p* = 0.27). An Ultrasound pleural scanning was systematically performed before a puncture for 43% of the responders (36% hepatogastroenterologists vs 70% pulmonologists; *p* < 0.001). A chest X-ray was performed before a puncture for 73% of the respondeurs (79% hepatogastroenterologists vs 54% pulmonologists; *p* < 0.001). In case of a spontaneous bacterial empyema, an albumin infusion was used by 73% hepatogastroenterologists and 20% pulmonologists (*p* < 0.001). A drain was used by 37% of the responders (37% hepatogastroenterologists vs 31% pulmonologists; *p* = 0.26).An Indwelling pleural catheter was used by 50% pulmonologists and 22% hepatogastroenterologists (*p* < 0.01). TIPS was recommended by 78% of the responders (85% hepatogastroenterologists vs 52% pulmonologists; *p* < 0.001) and a liver transplantation, by 76% of the responders (86% hepatogastroenterologists vs 44% pulmonologists; *p* < 0.001).

**Conclusions:**

The results of this large study provide important data on practices of French speaking hepatogastroenterologists and pulmonologists; it appears that recommendations are warranted.

## Introduction

The Hepatic hydrothorax (HH) is defined by the presence of a pleural effusion of a transudative nature greater than 500 ml secondary to some portal hypertension in a cirrhotic patient, in the absence of any causes of cardiopulmonary or malignant origins [1]. The HH occurs in 5% to 15% of patients with portal hypertension and cirrhosis and is associated with a significant mortality rate. The poor tolerance of the HH makes its therapeutic management difficult, which can be a source of iatrogenic complications [1–4]. The treatment of HH is mainly determined by whether or not a liver transplantation is feasable [2, 5, 6]. The mechanism of the HH formation is related to a unidirectional transfer of abdominal ascites to the pleural cavity through diaphragmatic breaches [4]. These breaches range in size from 0.03 to 6 mm in terms of diameter [5]. We can observe a unidirectional passage of ascites formed on the surface of the liver from the peritoneal cavity to the pleura under a hydrostatic pressure gradient, and the HH will surface when the accumulation of ascites in the pleural space exceeds the resorptive capacities of the pleura [7]. This mecanism leads to different treatments possibilities. Although there exists an extensive literature [1] on the hepatic hydrothorax and a recent very complete published review [8], there are no established and internationally recognized recommendations on the modalities of diagnosis and therapeutic management. The aim of this international multicenter French-speaking study conducted among pulmonologists (PN) and/or hepatogastroenterologists (HG) was to evaluate the knowledge and practices of physicians regarding the hydrothorax diagnosis and management in order to lead secondary to the possibility of establishing scientific recommendations.

## Methods

### Study design

#### Participating practitioners

This international francophone survey was conducted prospectively among French-speaking HG or PN practitioners in university hospitals (UH), general hospitals (GH), and private clinics in several francophone countries. This observatory survey was conducted under the aegis of the CFHTP, the SPLF and relayed by AFEF, SNFGE, ANGH, CREGG, SAHGE, SOBUHGEED, SBHGE, CHUM and the BASL, and from P from other countries.

The knowledge of the practitioners and their practices regarding the HH were assessed by means of a Google questionnaire. This questionnaire had been previously established before the survey by 12 HG and PN coordinators. The synopsis explaining the modalities of the study was sent, with reminders by the learned societies and coordinators over a period of 5 months from July 1st to November 30th 2020. The answers to the open-ended questions were analysed by two independent operators. The following data were analysed: the age, gender, type of practice, country of practice, experience of the responder, predominant specialty ie. PN vs HG, number of patients with HH followed by the responders. The questionnaire included the definitions and diagnoses modalities of the hepatic hydrothorax, including imaging procedures, thoracic scanners, pleural fluid analysis, modalities of therapeutic thoracocentesis: use of chest RX, thoracic ultrasounds, corrections of homeostasis abnormalities and use of albumin infusions. Some questions concerning the diagnosis and treatment of the pleural empyema were also noted. Finally, the modalities of the HH treatment with possibilities of treatments such as undwelling pleural catheter, talcage pleurodesis, drainage, TIPS and liver transplantation were determined. Some questions regarding the hydrothorax, the location of the hydrothorax, the possibilities of a hydrothorax in the absence of ascites, the diagnostic modalities to correlate the hydrothorax or a portal hypertension, the modalities of a therapeutic pleural puncture, the use of albumin, ultrasounds and chest X-rays, the modalities of a diagnosis and treatment of the empyema and a treatment of the hepatic hydrothorax are listed in Appendix 1 (from question A to question F6). The practitioners responding to the study were asked to answer the questions regarding the diagnosis and forward their answers in an email to the primary coordinator (JFC) to be included in the end-of-page signatory list. All these data were collected in an anonymous manner. The physician statistician (TLM) collecting the data was not informed of the names of the responders nor of their specialty.

The statistics are presented for qualitative values in proportion and mean (quantitative variable). Some comparative tests between the PN and HG were performed by Student’s test, Qui2 test or 5% alpha risk respectively.

We did not need an ethic committee for this study since it does not include patients. The Participants were all physicians who of course accepted to answer the questions from the survey.

## Results

**Fig. 1:**
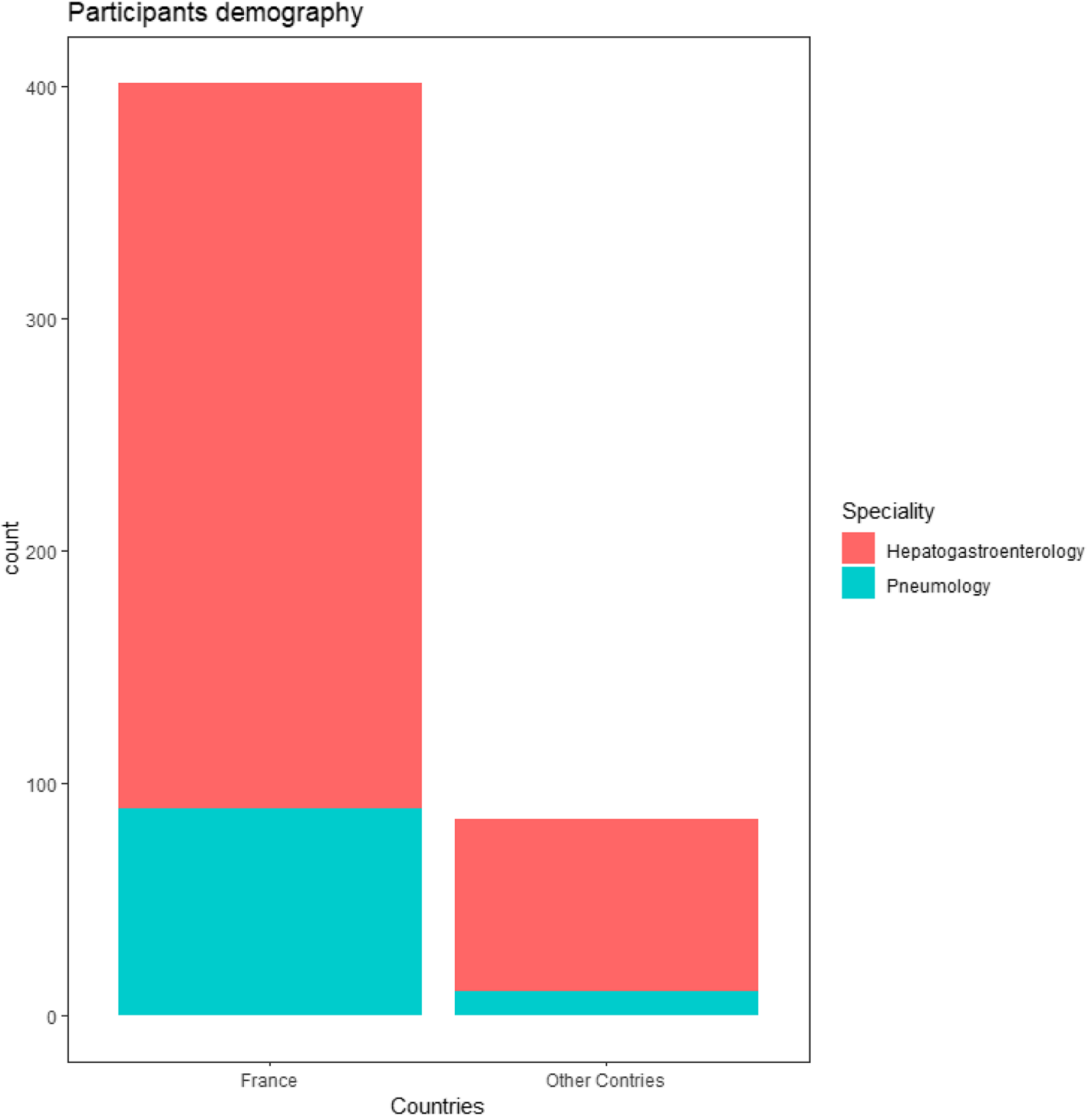
Participants demography
  Out of 1350 practitioners surveyed, 528 responded (39%).
  Among the 528 responders, 80 % were for France and 20% from 12 other countries: Algeria, Belgium, Benin, Burkina Faso, Congo Kinshasa, Ivory Coast, Lebanon, Morocco, Montreal, Romania, Switzerland, Tunisia.
  75% of the responders were HG, 20% PN, 5% had other specialties.
  The mean age of the responders was 40.6 years (SD 12.5), 44% were women.
  The majority of the responders (63%) works in academic centers and among the practitioners 77% were senior.
  The number of patients seen per practitioners in the year ranged from 0 to 60 (mean = 5.4 ; sd = 6.6 patients). The number of patients followed by HG was significantly higher than the number of those followed by PN (5.7 vs 4.1 patients, *p* = 0.01).
- B - Definitions and diagnosis of HH
  - B1 - Can a pleural effusion in a cirrhotic patient be related to portal hypertension?
    98% of the responders thought that pleural effusion in a cirrhotic patient can be related to portal hypertension (without any difference between whether the answers originated from HG or PN).
  - B2 - Can a hepatic hydrothorax be located only in the left lung?
    64% of the responders answered that the hydrothorax can be located in the left lung.
  - B3 - Can a hepatic hydrothorax exist in the absence of clinical ascites? Can a hydrothorax exist in the absence of ascites on theabdominal ultrasound?
    66% for HG / 57% for PN *p* = 0.25. (Fig. 2). 91% of the responders agreed on the fact that the hepatic hydrothorax may exist in the absence of clinical ascites (93% of the HG thought so compared to 80% of the PN, *p* = 0.25.) (Fig. 3). 60% of the responders were aware that the hydrothorax may exist in the absence of ascites on the abdominal ultrasound, 62% HG vs 25% PN, *p* = 0.54.

- A - General - demography - participants (Fig. 1)

**Fig. 2:**
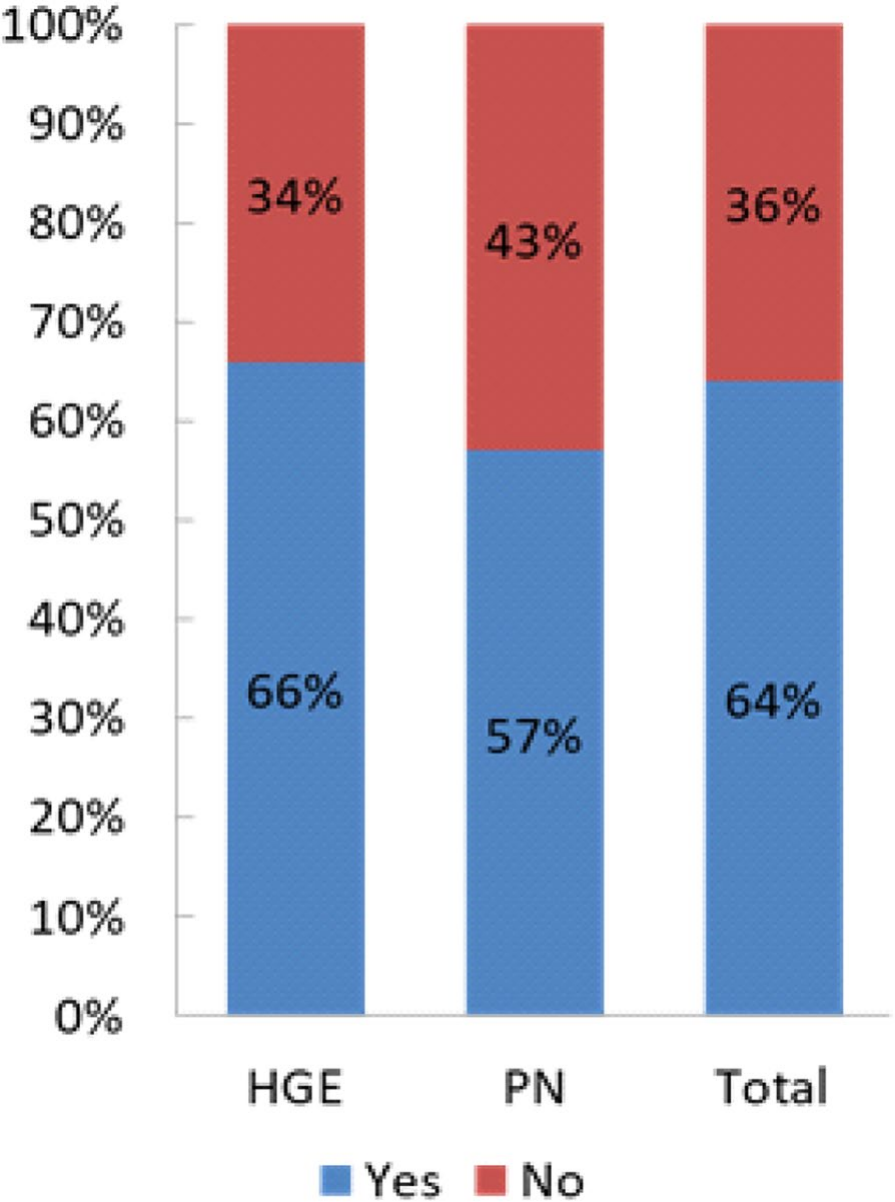
Left location of the hydrothorax

**Fig. 3:**
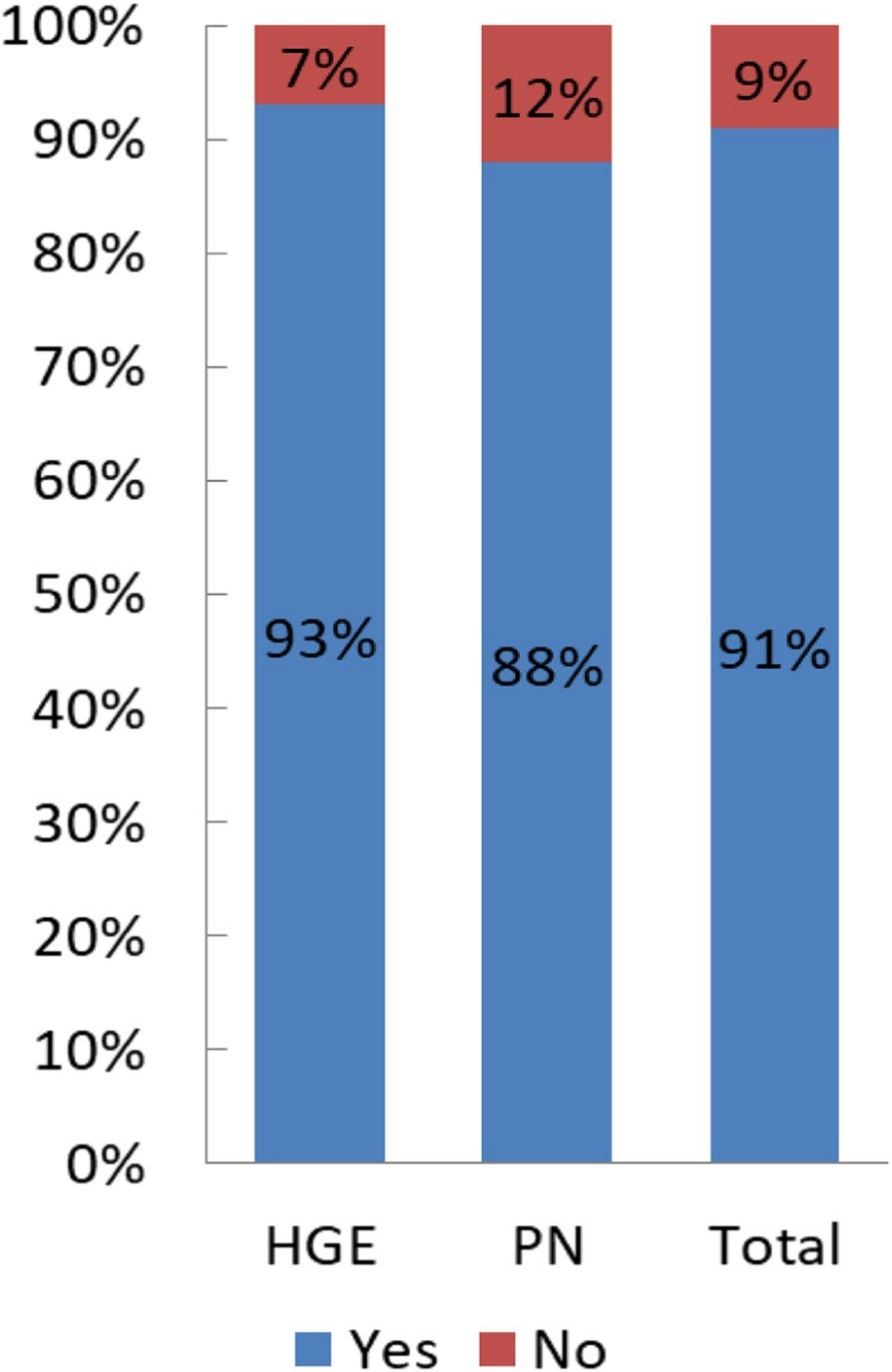
HH present in the absence of clinical ascites
  - B5 - Regarding the diagnostic modalities, what tests do you resort to in order to determine the origin of the pleural effusion?
    A pleural fluid examination with chemical dosages (total protein, albumin and LDH levels) and cytobacteriological examination was performed by 78% of the responders, a thoracic computed tomography by 41 % of them, a cardiac ultrasonography by 27% and a peritoneal scintigraphy by 8% of responders without any difference whether the answers originated from HG or PN.
- C - Complications of the hepatic hydrothorax
  The potential complications of the hydrothorax are shown in Table 1. Pulmonologists were more aware of the possibility of a tamponade (related to a cardiac compression with a ventricular collapse): 53.7 vs 30.6% for HG : *p*< 0.001 in patients with HH.

**Table 1:** Complications of the HH

Possible complications	Responses
Coughing	494 (94%)
SPE	504 (96%)
Dyspnea	522 (99%)
Respiratory distress	506 (97%)
Pleural mesothelioma	17 (3%)
Tamponade	188 (36%)
Hemoptysis	53 (10%)
  A large majority of the responders answered that coughing, a spontaneous bacterial empyema, dyspnea and respiratory distress can complicate the HH.
- D - Therapeutic pleural puncture (Fig. 4)
  - D1 - In which clinical situations would you suggest an evacuating pleural puncture?
     70.7% of the responders performed a pleural puncture in a case of dyspnea, 3.9% in a case of hemodynamic decompensation and 10% in a case of large effusion with no difference between whether the answers originated from HG or PN.
  - D2 - Before starting a therapeutic pleural puncture, do you correct the hemostasis? always, sometimes, or never. If yes, what do you prescribe?
    Before starting a therapeutic pleural puncture, 17% of the responders answered they never correct the homeostasis abnormalities and 82% answered they correct it (there was no significant difference between the answers originated by HG and PN). 50% of the responders used fresh frozen plasma (HG 55% vs PN 28%, *p*< 0.001) and in a case of thrombopenia 82% used a platelet perfusion without any significant difference between the answers originated by HG and PN.
  - D3 - Who usually performs the pleural punctures? A pulmonologist, radiologist, hepatogastroenterologist, junior or senior?
    The pleural puncture was performed in 50% of the cases by PN, in 38% of the cases by HG, in 7% by a radiologist and by other specialists in 4% of the cases with no difference between senior and junior.
  - D4 - Do you use an ultrasound scan before performing a pleural puncture?
    An ultrasound identification (pleural ultrasound) was routinely performed before initiating the pleural puncture by 43% of the responders, 36% of whom were HG and 70% PN, *P* < 0.001. (Table 2 and Fig. 4).

**Table 2:** Imaging examinations accompanying the puncture

Questions	Global	HGE	PN	*p* value
Pleural ultrasound before puncture	43%	36%	70%	< 0.001
Chest X-ray before puncture	73%	79%	54%	< 0.001
Chest X-ray after puncture	74%	77%	64%	0.005
  - D5 - Do you routinely send pleural puncture fluid for analysis? If yes, which tests do you request?
    35% of the responders answered they sent pleural fluid for analysis. The pleural fluid was examined for total protein, albumin and lactate dehydrogenase (LDH) levels, cell count Gram stain and culture in blood culture bottles by 95% of the responders without any significant difference whether the answers originated from HG or PN.
  - D6- Do you administer albumin after performing a pleural puncture ? - If yes, from which pleural volume subtracted and at which dosage?
    Human albumin infusion after performing a therapeutic pleural puncture was used by 60 % of the responders, HG 68% vs P 42% (*p* < 0.001).
    Human albumin infusion was generally used from 3 litres subtracted at the rate of one vial of albumin (8g/ litre) / 3 litres of pleural effusion subtracted (85% of HG vs 50 PN, *p* < 0.01).
  - D7 - Do you systematically perform a chest X-ray before initiating a pleural puncture? Do you perform a chest X-ray after initiating a pleural puncture?
    A Chest radiography was routinely performed before the pleural puncture by 73% of the responders, 79% HG / 54% PN, *p* < 0.001 (Table 2).
    A Chest X-ray was performed after a pleural puncture by 74% of the responders, 97% HG / 64% PN (*p* < 0.001) (Table 2).
  - D8 - When the patient presents ascites and a symptomatic hepatic hydrothorax at the same time, what strategy do you adopt? ascites puncture alone, pleural puncture alone, ascites and pleural puncture at the same time, ascites puncture followed by a pleural puncture if a symptomatic hydrothorax persists?
    In case of ascites and HH, 4% of the responders performed ascites puncture alone, 4% of them performed pleural puncture alone, 83% performed ascites puncture alone, then the responders answered they performed a pleural puncture in case the symptomatic hydrothorax persists with no significant difference between the answers originated by HG and PN, notably 8% of the PN performed a pleural puncture alone vs 2% of the HG (*p*=0.008).
  - D9 - Which ones of the following complications secondary to a thoracentesis have you experienced more often: hemothorax, pneumothorax, renal failure, vagal malaise, a vacua oedema, others?
    Regarding the complications related to the pleural puncture ; 90 % of the responders have encountered complications after a pleural puncture: hemothorax : 27% (HG 30% vs PN 17%, p=0.01), pneumothorax : 65% (without any significant differences), renal failure : 8% (HG 9% vs PN 3%, *p* = 0.07), vagal malaise:30% (HG 23% vs PN 55%, *p* < 0.001), a re-expansion pulmonary oedema : 13% (HG = 11% vs PN:21%, *p* = 0.02).

**Fig. 4:**
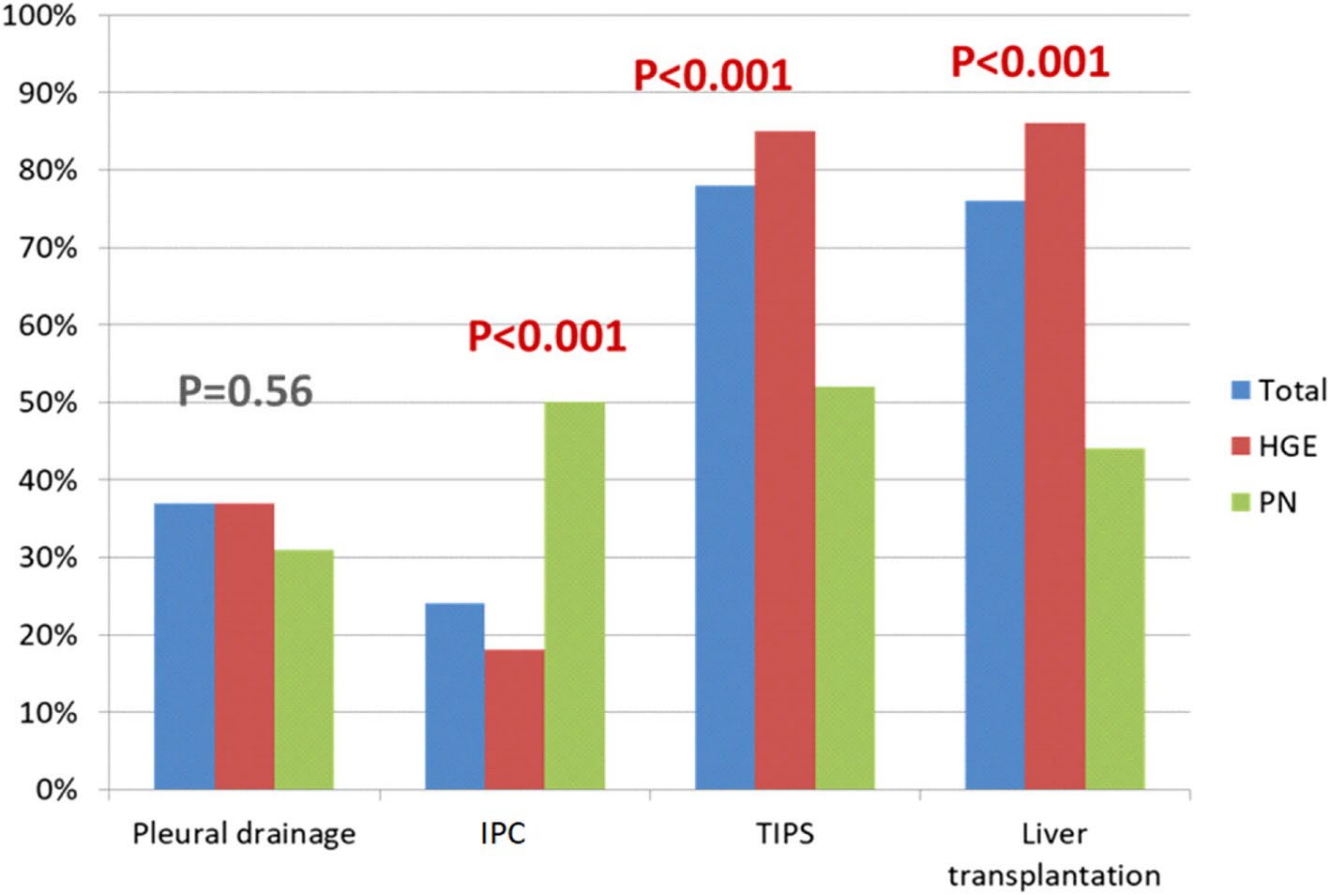
Treatment of « refractory» hydrothorax
- E - Spontaneous bacterial empyema (SBE) of HH
  - E1 - What is the definition of a bacterial empyema? Can a bacterial empyema occur in the absence of a spontaneous infection of the ascites fluid?
    Regarding your definition of a bacterial empyema, a correct question according to the literature data was obtained by 15% of the responders, 17.6% of HG, 5.6% for PN, *p* = 0.0032. Considering the possibility of a SBE in the absence of SBP ; 78% of the responders confirmed that this was possible, 5% did not confirm and 17% did not know. HG :81% PN 69%, *p* = 0.03).
  - E2 - If a bacterial infection occurs: Do you use the same antibiotics as for a spontaneous ascites fluid infection? If you answered yes to the previous question: according to a duration equivalent to the SBP?
    In case of a spontaneous bacterial empyema, 78 % answered that they used the same antibiotics as for the SBP and the use of that same antibiotic was more frequent for HG ,78% vs 48% of PN ; *p* < 0.001.
    The duration of the antibiotic therapy was the same as for the SBP for 68% HGE vs 38% PN. *p* < 001.
  - E3 - If the quantity of fluid allows it, do you perform a control puncture 48 hours afterwards to check the polymorphonuclear count?
    53% of HGvs14% PN ; *p* < 001 performed a control puncture to check the polynuclear count after 48 hours if the quantity of fluid allowed it.
  - E4 - Do you administer albumin? And if so, how? Do you use the “Sort protocol”? Are there any clinic-biological parameters that would prompt you to prescribe albumin?
    Albumin administration was used to fight a pleural fluid infection by 66% of R, 73% HG, 20% PN (*p* < 0.001) and albumin infusion was performed according to the "Sort" protocol by 80% of the HG / 45% of PN; *p* < 0.001. No alternative use of albumin was quoted.
    Are there clinic-biological parameters that would prompt the responders to prescribe albumin? These parameters were used by 47 % of the responders whereas 53% did not use specific parameters, without any significant differences between the answers originated by HG and PN. The responders took into account the low albumin level for 18% of them (at the level of 25 g/L) and 20% of the responders took into account the renal insufficiency (at the cut off of 133 µmol/l).
- F - Treatment of the hepatic hydrothorax (Fig. 4)
  - F1 – Do you agree that the treatment of HH is based primarily on a low salt diet and diuretics
    93% HG and 84% PN *p* < 0.006 consider that the treatment of hepatic hydrothorax relies primarily on the combination of a low salt diet and diuretics.
  - F2 - After how many pleural punctures performed over a period of two months will you consider starting another type of treatment?
    25% of the responders considered an other type of treatment after 2 pleural punctures performed during a 2 months period, 25 % after 3 and 32 % after 4 (median ; 2-6) without any differences between the answers originated by HG and PN.
  - F3 - Is there a consensus definition of a refractory HH, what do you think it is?
    44.5 % HG thought that there was no definition of a refractory hepatic hydrothorax, vs 30.2 % PN ; *p* < 0.04.
  - F4 -  In case of a refractory hydrothorax, do you discuss the case collegially? and if so, with which specialists?
    96% of the responders answered they would discuss collegially the therapeutic-options of a refractory hydrothorax with other specialists
    The collegial discussion includes HG, PN, thoracic and hepatic surgeons for 97% of the responders without difference between the answers originated by HG and PN.
  - F5 - The last question concerned the treatment of a recurrent hydrothorax. Do you use pleural drain, talcation, IPC, TIPS, liver transplantation and according to which criteria: age, gender, Child pugh, score, MELD score or others.
    38 % of the responders answered they used pleural drainage without any significant difference between the answers originated by HG and PN (Fig. 4), talcage (51% PN vs 31% HG, *p* < 0.001), in the opposite, IPC was used by 50 % PN and 22 % HGE (*p* < 0.001), TIPS by 84 HG and 77 % PN ; *p* < 0.001 and liver transplantation by 85 % HG and 76 % PN *p* < 0.001 (Fig. 4).
    The criteria selected to determine the optimal treatment were essentially the age at the level of 65 years for 26% of the responders, Child Pugh score for 24%, MELD score for 17% and the possibility of a liver transplantation for 16%, TIPS possibility for 7 % and hepatic encephalopathy for 3 %(183/528 gave no responses). The responders favoured TIPS when it was feasible and when there was no other indication than the HH for the the liver transplantation, IPC was mainly used while waiting for the transplantation and in case of contraindication to a transplantation and/ or to TIPS.

## Discussion

### General data

In this large international French-speaking multicenter study, we reported the results of the responses of 528 hepatogastroenterologists or pulmonologists working in Europe and outside Europe (in academic and non academic hospitals, or private practices, senior or junior). We were thus able to evaluate the knowledge on the diagnosis and therapeutic management of the hepatic hydrothorax in cirrhotic patients within a wide range of clinicians.

While many reviews have been written on the hydrothorax [1–7] and a recent complete extensive published review on the topic of the hydrothorax management in 2020 [8], to our knowledge, no practice survey has yet addressed this topic in either the hepatology or pneumology literature. The response rate of 39% in this international study is very significant. Thus, our study appears to be representative of a large panel of hepatogastroenterologists and pulmonologists practicing either in France or in other French-speaking countries outside France. Nevertheless, it should be noted that there is a relative under-representation of the responses from PN, even though they were solicited by the Société Pneumologique de Langue Française which is an international society which gathers together all the French-speaking countries involved in pneumology. This low representation could be explained by a certain unfamiliarity from these specialists regarding the hepatic hydrothorax management since they are less involved (see results) in taking care of these patients.

One of the limitations of this survey is that the answers to the survey questions aim at treating the patients. For this reason, there may have been some intermediate responses among the practitioners regarding their best intentions and common practice, especially since there are no formalised recommendations for the management of the hydrothorax.

It should also be noted that the number of practitioners participating in this study was much higher than the one of two national French studies [9, 10], This rate is much higher than the one of the practice study on the use of growth factors in patients with hepatitis C treated with Interferon which averages 30% of responders [9] and than the one of the second study aimed at prophylactic antibiotic therapy use in a large panel of French university and non-university practitioners where the response rate was of 30% [10].

Our response rate was slightly lower than the 45% response rate of the study on albumin coordinated by one of us (JFC) which brought together several societies: AFEF, ANGH, CREGG [11]. Nevertheless, our response rate remained very important if we consider how barely known the hydrothorax still is and the absence of international recommendations. This satisfactory response rate can be explained by:

1. the anonymous and scientific nature of the questionnaire,
2. the personal invitation to answer to this survey from several French or non-French hepatological or pneumological learned societies.
3. numerous reminders made by the coordinators through the learned societies, and also individually.

### General characteristics of the hydrothorax—complications—therapeutic pleural punctures

The possibility of a hydrothorax related to a portal hypertension (HH) was well known by nearly 99% of the responders without any difference whether the answers originated from HG and PN and most of the responders knew that on the one hand, a pleural effusion can be related to a portal hypertension and on the other hand, that a hydrothorax can be located only in the left lung (see Fig. 2).

The responders were fairly well aware of the possibility of a hydrothorax in the absence of clinical ascites.

However, regarding the possibility of the existence of a HH in the absence of ascites on the abdominal ultrasound, only 60% of the responders answered positively to this question without any difference whether the answers originated from the HG **or** the PN, although this possibility is well known in the literature [3, 7].

In the absence of clinical ascites, the majority of the responders performed a pleural puncture with cytobacteriological, cultures and a biochemistry examination of the pleural fluid. Almost no responders knew about the existence of the peritoneal scintigraphy, which is however the essential examination to make the diagnosis of a hydrothorax in the absence of clinical ascites [12].

Most of the pleural punctures were performed by the PN. While the risk of hemothorax and pneumothorax was increased [13] after repeated therapeutic pleural punctures and the risk was increasing with the number of punctures in multivariate analysis [8, 13], few practitioners performed an ultrasound before the puncture in order to avoid a pneumothorax. It is also relevant to underline that the occurrence of complications during the pleural puncture was also associated with a risk of future complications; *P* < 0.01 [13, 14] but this element was not assessed in our survey. It has been established [8, 13] that a platelet count of less than 50 000 and an elevated INR are independent predictive factors of a hemothorax. In accordance with these data, the majority of the responders of this study corrected homeostasis abnormalities by fresh frozen plasma or platelet administration when needed.

Albumin was used more often by HG than by PN. The results of albumin use among HG were roughly comparable to those of our previous survey on albumin use in France [11], where 70% of the responders used albumin without specifying the compensation volume. We must keep in mind that that the French experts [2, 3] recommend the use of albumin in case of a pleural puncture of 2 L or more for a hydrothorax. Most of the responders performed an ascites puncture alone followed by a pleural puncture in case of tense ascites, or in case of a symptomatic hydrothorax as it is recommended by most of the reviews [5–7].

### Spontaneous bacterial empyema

The correct definition of a SBE was rarely given no matter whether the responders were HG or PN. The knowledge on this topic was poor, with respect to its definition and particularly with respect to the number of polymorphonuclear cells required to make the diagnosis of a SBE [14, 15].

The possibility of a SBE without a concomitant SBP, which is possible in at least 40% of the cases [1, 8, 14], was relatively well known.

For most of the responders, the same antibiotics used as the ones used to fight the SBP were used for a comparable duration in the treatment of the SBE especially for the HG. Most of the practitioners did not control the polynuclear count in case of a possible pleural puncture after 48H, but it is to be noted that there were no questions about the possibility of controlling the associated ascites fluid in case of a concomitant SBP and /or SBE. As for the administration of albumin recommended by some authors [2], it was rarely used by any of the HG by analogy with the SBP using Sort's protocol in the majority of the cases.

The difference regarding the albumin administration was highly significant between the HG and PN, human albumin infusion being primarily used by the HG.

Regarding the criteria for the albumin compensation in the case of an empyema, the most frequent answers found in our study were a low albumin level and the presence of a renal insufficiency.

These very imperfect elements of answers are to be taken into consideration because the SBE associated with a hepatic hydrothorax is a serious complication, source of sepsis, septic shock, multi-visceral failure and of a significant mortality rate [8, 14–17].

Thus, we acknowledge that precise recommendations concerning the diagnosis of the SBE associated with a cirrhosis and its treatment modalities are highly necessary.

### Treatment of the hepatic hydrothorax

#### Low salt diet—diuretics

Regarding the treatment of the hydrothorax, most of the responders answered that the treatment of hepatic hydrothorax relies primarily on the combination of a low salt diet and diuretics. It should be noted, however, that our questionnaire did not specify whether a combined use of furosemide and spironolactone associated with a low-sodium diet was preferable to the use of only one diuretic whereas most studies [8] recommend the co-administration of furosemide 40 mg and spironolactone 100 mg per day to obtain a greater mobilisation of the hydrothorax, with a stepwise increase in this combination of diuretics [8, 18].

20 to 30% of the patients may have a persistent or recurrent hydrothorax despite the gradual and well-conducted use of diuretics and a low salt diet [8], however there was no clear definition of what a refractory hydrothorax is for 45% of the responders.

Furthermore, the use of diuretics can lead to hydro-electrolytic disorders, renal failures, hemodynamic instability for fragile patients [8].

The number of repeated pleural punctures that should lead to the discussion of other treatments was in median 4 without significant difference between the answers originated by the PN and the HG. This number is high due to the risk of developing a pneumothorax and hemothorax as stated above [8, 13].

Most responders recommended a collegial discussion among the HG, PN, liver and thoracic surgeons for patients with difficult -to -treat HH.

#### Pleural drain

Although several publications have shown that a pleural drain placement for a hydrothorax was associated with a high rate of complications [19]; it has been observed that it was performed by 38% responders in this study.

In a recent study [20], it was reported that the in-hospital mortality after a pleural drain placement for a hydrothorax occurred for 40% of cirrhotic patients with a cirrhosis classified as Child Pugh (CP) C and 16% of stage B patients.

Moreover, in a very important recent study [21] carried out on more than 140,000 cirrhotic patients, among whom 1,981 presented a HH requiring repeated pleural punctures, 905 patients had a pleural drain placed inside them and for these patients the length of hospitalisation and the mortality rate were twice as high as for patients with a simple thoracic resection [21].

Thus,for all these reasons, the AASLD recommendations state that a pleural drain placement is contraindicated for patients with a hepatic hydrothorax [22].

#### Talcage pleurodesis

The Talcage pleurodesis was used essentially for metastatic hydrothorax effusions, and was mainly used by pulmonologists in the present study.

The studies using bleomycin, biomycine, aminocycline, showed that the success rate of the pleurodesis in 8 studies reported in the general study [23, 24] varied from 47 to 75% with a recurrence rate requiring a repeated puncture in 25% of the cases.

However, the results concern a small series with an overall effectiveness of 44% going up to 60% when a closure of the breaches under a videothoracoscopy is associated [23, 24].

It should be noted that when a videothoracoscopy was performed, a rate of 75% of responses was observed but this possibility was not assessed in our survey.

#### Indwelling catheter

The IPC is a fenestrated catheter that is inserted and tunnelled percutaneously into the pleural space to allow an intermittent drainage and facilitate a pleurodesis.

It should be noted that in our study, the IPC was used mostly by PN.

Over the past decade, the tunnelled catheter has shown great benefit in the management of malignant (metastatic) pleural effusions. It was adopted in this indication by the FDA in 2017 [8].

Several studies have shown a spontaneous pleurodesis after the placement of the IPC. Nevertheless,in the majority of the studies, the patients also received a liver transplant [8, 25–27] and the IPC serves as a bridge to the liver transplantation.

Thus to date, the rate of spontaneous pleurodesis attributed to IPC is probably overestimated. The main studies have been analysed recently by Banini et al. [8].

In a prospective study of 24 patients who received an IPC [25], an effective pleurodesis was observed and the catheter removal was possible for 33% of the patients with a mean time of 131 days to reach the pleurodesis.

In another retrospective study conducted among 62 patients, 53% of them (33 of 52) were able to wait for a liver transplantation. In this study, 9 patients (14%)developed a spontaneous empyema after a period of 180 days and were subsequently able to receive a liver transplant [26].

Finally, in a recent multicenter retrospective study conducted among 79 patients from 8 medical centers, IPC were placed in 21 of them (27%) as a bridge to a liver transplant, and the remaining 58 of them (73%) for palliation. Eight patients (10%) developed a pleural space infection; 2 (2,5%) died consequently to a catheter-related empyema and sepsis. In the cohort, an older age was predictive of mortality on multivariable analysis [27].

The IPC, which appears to be an interesting palliative treatment of a symptomatic hydrothorax awaiting for a liver transplantation, should be more widely evaluated [8]. Our participants answered that the IPC was mainly used in this study as a transitory device while waiting for a transplantation and in case of contraindication to a transplantation and /or to TIPS.

### Other treatments

It should be noted that in the methods leading to the prevention of fluid transfer into the pleural space, our questionnaire did not evaluate the following treatments: continuous pressure ventilation CPAP nor the repair of diaphragmatic breaches because these procedures have been little evaluated so far and are not used as a common practice in the countries participating in the study. This is clearly a limitation in our study. This procedure is still a very marginal practice in France. Other treatment possibilities (reviewed in 8) were not assessed in our survey.

### TIPS

In the treatment of a refractory hydrothorax, most of the responders resorted to TIPS in the absence of contraindications in the treatment of a refractory hydrothorax. The difference in the answers was highly significant between the PN and the HG and these ones were more aware of this technique.

Numerous studies and a recent meta-analysis showed the interest of TIPS in the management of a refractory hydrothorax [8, 28–33].

Although the number of studies related to the treatment with TIPS is limited, the use of a TIPS leads to an improvement with a disappearance of the pleural effusion or a reduction of it with a sensitivity to diuretics in 60 to 90% of the cases [8].

In a large retrospective study of 40 patients with a CP score B or C and a refractory hydrothorax, 90% of the patients who underwent a treatment with TIPS showed an improvement of the hydrothorax with a complete resolution in 70% of the cases. In this study, the 1-year surveillance rate was of 64% but 50% of the patients developed a TIPS malfunction with the need for a TIPS revision in 90% of the cases [31].

Recently, a meta-analysis of 6 studies related to TIPS use for a refractory hydrothorax has been published [33]. This study which included 198 patients showed that the procedure resulted in the resolution of the hydrothorax in 73% of the cases, a complete resolution in 56% of the cases and a partial one in 7% of the cases. The average follow-up time was 10 months.

Up to 15–25% of the patients could develop a hard-to-control hepatic encephalopathy [8]. Nevertheless, it has recently been shown that covered TIPS preceded by the administration of rifaximine and lactulose would decrease the incidence of post-TIPS encephalopathy [34].

A pre-therapeutic workup is important before deciding on performing TIPS and it is usually done during a discussion with a liver transplant team. In our survey, the responders favoured TIPS when it was possible and when the patient had no other indication than a HA for a liver transplantation.

Finally, the patients who can benefit from TIPS do not preclude the need for a liver transplant.

### Liver transplantation (LT)

LT is the definitive treatment for a refractory hydrothorax [8, 35, 36]. This treatment was known by 78% of the responders and more frequently by the HG. A liver transplantation was the treatment chosen by our responders especially if there was another indication for a transplantation in the presence of the hydrothorax. A large MELD, a history of ascites fluid infection or a spontaneous bacterial empyema should be referred to a LT center [8]. In this regard, it is important to note that the collegial discussion when treating a refractory hydrothorax included a liver transplant team for our responders.

The survival rate after a LT in patients with a hydrothorax is not different from that of other patients, 70% after 8 years. In a recent study, the LT was the key element to a short- and long-term survival after a hydrothorax [35, 36].

The mortality rate after 1 to 3 years reached 77% in non-transplanted patients and 21% in transplanted patients [36].

## Conclusions

Although our large survey shows some weaknesses because of the nature of the study and the absence of questions on other therapeutic possibilities largely reviewed recently [8], it added significant data regarding the knowledge of the HG and the practitioners concerning the diagnostic modalities and therapeutic management of the HH. The Hepatic hydrothorax is not known enough, the scientific recommendations according to the age, liver function tests and possibilities of TIPS and/or liver transplantations should be established. Our team is currently working on the possibility of presenting such recommendations.

## Data Availability

Not applicable.

## Appendix

### Questions of the doodle form

- A - Demographic data.
  General - demographics - participants.
  The following parameters were noted:
  The Age, gender of the participants, type of practice, location of practice : University Hospital or equivalent outside France, General Hospital or equivalent outside France, Private practice or mixed, country of practice. Predominant specialty: hepatogastroenterology, pneumology or other specialities were also noted as well as with the status of the participants: resident or equivalent of 1st or 2nd year, resident or equivalent of 3rd year, senior of 5 to 10 years of experience, senior of 11 to 15 years of experience, senior of more than 15 years of experience. The number of different patients with a hepatic hydrothorax seen in the year by each practitioner was also noted.
- B - Data concerning the definitions and diagnoses of a hepatic hydrothorax were reported.
  The questionnaire included the definitions and diagnoses of a hepatic hydrothorax with the following questions:
  - B1 - Can a pleural effusion in a cirrhotic patient be related to a portal hypertension?
  - B2 - Can a hepatic hydrothorax be located only in the left lung?
  - B3 - Can a hepatic hydrothorax exist in the absence of clinical ascites? - Can a hydrothorax exist in the absence of ascites on the abdominal ultrasound?
  - B4 - Regarding the diagnostic modalities, what tests do you use to determine the origin of the pleural effusion?
- C - Complications of a hepatic hydrothorax
  Can a thoracic hydrothorax lead to one or more of the following complications: cough, pleural fluid infection, dyspnea, respiratory distress, mesothelioma, tamponade, hemoptisis.
- D - Therapeutic pleural puncture
  - D1 - In which clinical situations would you suggest an evacuating pleural puncture?
  - D2 - Before a therapeutic pleural puncture, do you correct the hemostasis? always, sometimes, never? If yes, what do you prescribe?
  - D3 - Who usually performs the pleural punctures? A pulmonologist, radiologist, hepatogastroenterologist.- Is it usually done by a senior or a junior?
  - D4 - Do you use an ultrasound scan before performing a pleural puncture?
  - D5 - Do you routinely send the pleural puncture fluid for analysis? If yes, which tests do you request?
  - D6- Do you administer albumin after a pleural puncture ? - If yes, from which pleural volume subtracted and at which dosage?
  - D7 - Do you systematically perform a chest X-ray before initiating a pleural puncture? Do you perform a chest X-ray after a pleural puncture?
  - D8 - When the patient presents ascites and a symptomatic hepatic hydrothorax at the same time, what strategy do you adopt? ascites puncture alone? pleural puncture alone? ascites and pleural puncture at the same time? ascites puncture followed by a pleural puncture if a symptomatic hydrothorax persists?
  - D9 - Which of the following complications secondary to a pleural puncture have you experienced more often: hemothorax, pneumothorax, renal failure, vagal malaise, a vacuo edema, others?
- E - Spontaneous bacterial empyema
  - E1 - What is the definition of a bacterial empyema? Can a bacterial empyema occur in the absence of a spontaneous infection of the ascites fluid?
  - E2 - If a bacterial infection occurs: Do you use the same antibiotics as the ones you use to fight a spontaneous ascites fluid infection? If you answered yes to the previous question: according to a duration equivalent to the SBP?
  - E3 - If the quantity allows it, do you perform a control puncture within 48 hours to check the polynuclear count?
  - E4 - Do you administer albumin? And if so, how? Do you use the “Sort protocol”? Are there any clinico-biological parameters that would prompt you to prescribe albumin?
- F - Questions aimed at evaluating the treatment of the HH
  - F1 - Do you agree that the treatment of the HH is based primarily on a low salt diet and diuretics?
  - F2 - After how many pleural punctures performed over a period of two months will you consider another type of treatment?
  - F3 - Is there a consensus definition of a refractory HH, what do you think it is?
  - F4 - In the case of a refractory hydrothorax, do you discuss the case collegially? and if so, with which specialists?
  - F5 - The last question concerned the treatment of a recurrent hydrothorax. Do you use pleural drain, talcation, IPC, TIPS, liver transplantation and according to which criteria: age, gender, Child pugh, score, MELD score or others.

## Abbreviations

HH: Hepatic hydrothorax
HG: Hepato-gastroenterologist
PN: Pulmonologist
TIPS: Trans-jugular intra hepatic porto-systemic shunt
UH: University hospitals
GH: General hospitals
SBE: Spontaneous bacterial empyema
SBP: Spontaneous bacterial peritonitis
CP: Child Pugh score
IPC: Indwelling pleural catheter
LT: Liver transplantation
CFHTP: Club Francophone d'Hypertension Portale
SPLF: Société Pneumologique de Langue Française
AFEF: Société Française d'Hépatologie
SNFGE: Société Nationale Française de GastroEntérologie
ANGH: Association Nationale des Hépato-Gastroentérologues des Hôpitaux Généraux
CREGG: Club de Réflexion des Cabinets et Groupes d'HépatoGastroentérologie
SAHGE: Algerian Society of Hepatogastroenterology
SOBUHGEED: Society of Hepatogastroenterology of Burkina Faso
SBHGE: Society of Hepatogastroenterology of Benin
CHUM: Centre Hospitalier de l’Université de Montréal
BASL: Belgium Society of Hepatology
